# Nitrogen-Doped Hollow Carbon Spheres-Decorated Co_2_SnO_4_/WS_2_ Heterostructures with Improved Visible-Light Photocatalytic Degradation of Organic Dye

**DOI:** 10.3390/molecules30092081

**Published:** 2025-05-07

**Authors:** Muthuraj Arunpandian, Tae Hwan Oh

**Affiliations:** School of Chemical Engineering, Yeungnam University, 280 Daehak-Ro, Gyeongsan 38541, Republic of Korea

**Keywords:** N-doped carbon spheres, heterostructure, degradation, affordable, EDX analysis, stability

## Abstract

Advanced photocatalytic materials for environmental cleanup need to be developed in response to growing concerns about water pollution. This paper presents a novel N-doped hollow carbon spheres (NHCSs)-supported Co_2_SnO_4_/WS_2_ heterostructure synthesized using a hydrothermal approach and examined using various characterization techniques to evaluate the crystal structures, functional groups, surface morphology, chemical properties, and optical characteristics. The photocatalytic performance of the Co_2_SnO_4_/WS_2_@NHCSs composite was assessed by degrading Congo red (CR) under visible light, resulting in a notable degradation rate of 87.22% in 60 min. The enhanced degradation efficiency is ascribed to the Z-scheme heterojunction charge-transfer mechanism, which augments sustained charge separation while suppressing recombination under visible-light irradiation. Furthermore, the quenching experiments revealed that specific superoxide radicals (^•^O_2_^-^) and hydroxyl radicals (^•^OH) were integral to the degradation reaction, and a potential Z-scheme charge-transfer pathway mechanism for the effective Co_2_SnO_4_/WS_2_@NHCSs photocatalysts was also suggested. The potential degradation mechanism was suggested using LC-MS analysis. This study highlights the promise of Co_2_SnO_4_/WS_2_@NHCSs composites for practical wastewater treatment applications, providing a sustainable and effective solution for environmental remediation.

## 1. Introduction

The urgent necessity for industrialization to fulfil the expanding human demand is brought about by the fast increase in the global population. The effects of industrialization on living things are hazardous, although they have improved human existence. The textile, food, plastic, pesticide, and other related industries are major polluters and emitters of organic dyes into the environment. Discharged organic pollutants and dyes pose a threat to aquatic life and the ecosystems around them, even in modest quantities, because of their toxicity [1,2]. Therefore, there is an urgent need to find efficient and cost-effective ways to remove toxic organic dyes from wastewater. Treatment of wastewater has been suggested using a variety of physical, chemical, and biological techniques, such as sorption, electrolysis, ion exchange, catalytic degradation, enzymatic and microbial breakdown, membrane separation, sedimentation, and more [3,4,5]. Since sorption simply changes the phase of the dye molecules, additional treatment is still necessary to eliminate harmful dyes from wastewater [6]. The use of chemical techniques is expensive since they rely on electrical energy [7]. Also, biological methods are not a good substitute because they cannot break down complex organic dyes [8]. Because of its environmentally benign approach and ability to eliminate secondary pollutants, photocatalytic degradation is becoming increasingly popular [9,10]. Toxic organic dyes can be oxidized to non-toxic components using this approach, which involves creating free radicals using light energy [11]. Photocatalysis is an effective technique for wastewater treatment, regarded as environmentally sustainable and economical due to its minimal energy demands and straightforward operational conditions, including ambient temperature and atmospheric pressure, enabling the degradation of organic pollutants without producing additional waste [12]. Photocatalytic processes entail the activation of a catalyst through radiation (either UV or visible), resulting in the formation of electron–hole pairs that interact with H_2_O, OH^–^, and O_2_ to produce oxidizing species (e.g., superoxide anion radical, singlet oxygen, and hydroxyl radical) responsible for the degradation of pollutants [13]. Catalysts must fulfil several criteria, including affordability, stability, high reactivity, and ease of recovery [14].

Titanium dioxide (TiO_2_) is a popular photocatalyst for the removal of organic dyes when exposed to UV radiation because of its physical and chemical properties. However, using visible light or sunlight to degrade these dyes is hindered by its wide bandgap, which is around 3.2 eV [15,16,17]. Thus, research is concentrated on discovering new catalysts that can degrade organic contaminants using sunlight and have a bandgap that extends from the visible to the near-infrared. Recently, semiconducting transition metal dichalcogenides (TMDCs) have garnered considerable interest as effective photocatalysts driven by visible light due to their narrow bandgaps. Transition metal dichalcogenides (TMDCs) are two-dimensional layered materials composed of X-M-X layers, where M represents a transition metal and X denotes a chalcogen. These layers are bound by robust covalent bonds, while the interlayers are linked by weak van der Waals forces, enabling facile exfoliation to monolayers. These materials are utilized in several domains due to their distinctive optical, electrical, and mechanical properties [18,19]. As photocatalysts, chalcogenide has a narrow-bandgap semiconductors have found extensive environmental application. Two-dimensional transition metal dichalcogenides (TMDs) such as WS_2_ have generated significant interest as model semiconductors. Tungsten disulfide (WS_2_) has been studied as an efficient photocatalyst capable of degrading various contaminants and eliminating pollution, attributed to its low bandgap (1.35–2 eV), extensive surface area, robust W–S bond, and broad spectral coverage. Tungsten disulfide has been investigated for its possible application as a photocatalyst, a substance that can enable the transformation of light energy into chemical energy. Tungsten disulfide can generate electrons and holes when exposed to sunshine or other light sources, facilitating the degradation of organic contaminants [20,21,22].

On the other hand, to overcome the limitations of selectivity and efficiency, numerous photocatalysts have been engineered and formulated [23]. Composite materials have lately garnered interest as adaptable catalysts for the rapid and efficient degradation of organic pollutants into innocuous and non-toxic compounds. Recent results indicate the use of Co_2_SnO_4_-based materials for the decomposition of industrial dye effluents. The catalytic efficiency of Co_2_SnO_4_ in photocatalysis is chiefly determined by the substantial rate of reabsorption of photogenerated electron–hole pairs. The surface morphology and crystalline structure are regarded as the principal determinants of declining performance. Cobalt-based bimetallic oxides have been shown to be effective in degrading organic contaminants. This results from the improved conductivity and electron transport of the catalysts, produced by the synergistic effects of redox couples among several metal species [24]. Cobalt-based nanocatalysts, specifically Co_2_SnO_4_, have several limitations including agglomeration, reduced specific surface area, and leaching of cobalt and tin. Therefore, to address these limitations, it is essential to disseminate these nanoparticles in appropriate supports [25,26].

To enhance the photocatalytic efficacy of the Co_2_SnO_4_/WS_2_ binary system, the hybrid nanostructure developed by incorporating carbon materials has received significant interest. Carbon spheres (CSs) are prevalent carbon materials often combined with semiconductors because of their superior electrical conductivity, thermal–chemical stability, extensive specific surface area, cost-effectiveness, and straightforward manufacturing [27,28,29,30,31,32]. Liu et al. [33] indicated that the CSs influenced the properties and photocatalytic efficacy of the CSs/g-C_3_N_4_ composites. The CSs/g-C_3_N_4_ composites exhibited enhanced photocatalytic efficiency for the breakdown of organic contaminants, including antibiotics and dyes. McEvoy et al. [34] synthesized Ag/AgCl-activated carbon composites, which demonstrated superior visible-light (VSL) photocatalytic efficacy for the destruction of methyl orange and phenol. These carbon compounds can function as a photosensitizer to inject electrons into the semiconductor, thus enhancing the separation of electron–hole pairs and facilitating the response to visible light. Consequently, the photocatalytic efficacy of photocatalysts is enhanced [33]. Especially, hollow carbon spheres (HCSs) surpass carbon nanotubes and two-dimensional materials owing to their numerous advantages, such as substantial void spaces, low density, and extensive specific surface area [35]. Their three-dimensional architecture facilitates efficient charge movement and improves storage capacity [36], indicating favorable application potential for energy storage devices. Nitrogen (N) doping in carbon-based materials can improve surface polarity, wettability, redox sites, and electrical conductivity [37,38]. Nitrogen-doped hollow carbon spheres (NHCs) are particularly promising carbon structures due to their unique hollow cavity-porous shell architecture, which enhances their potential applications in energy storage and conversion, adsorption, catalysis, and other domains [39].

Due to the disparate bandgaps and dimensions of Co_2_SnO_4_ nanocubes and 3D nanoflower WS_2_, the aggregation of Co_2_SnO_4_ nanocubes onto the surfaces of larger WS_2_ facilitates the formation of a van der Waals heterojunction at their interface, enabling rapid charge transfer inside the Co_2_SnO_4_/WS_2_ nanohybrids. Therefore, it is anticipated that the integration of NHSCs with Co_2_SnO_4_/WS_2_ nanohybrids to create a hierarchical heterostructure may serve as an effective technique for developing a stable, visible-light-responsive photocatalyst. To date, the synthesis of hierarchical Co_2_SnO_4_/WS_2_@NHCSs heterostructures and their subsequent application in photocatalytic environmental purification has not been reported.

Within the context of photocatalytic degradation, this research presents a unique application of heterostructure nanocomposites including Co_2_SnO_4_/WS_2_@NHCSs. These nanocomposites were manufactured using a hydrothermal approach. In this study, their structural, optical, and textural features are elaborated upon, and their efficiency in degrading tetracycline under conditions of visible light is evaluated. Furthermore, the research highlights the potential of these materials in the development of new water treatment technologies.

## 2. Results and Discussion

### 2.1. Crystalline Structure and Functional Group Analysis

**Figure 1:**
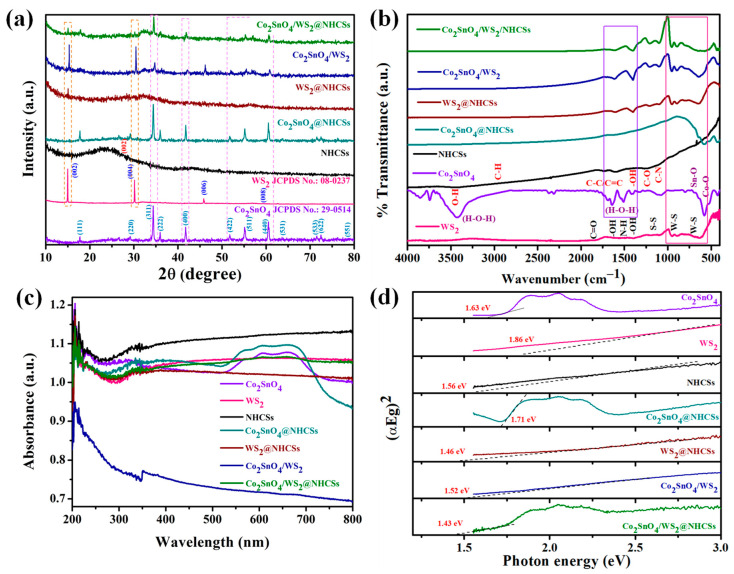
Crystal structure and functional group analysis: (**a**) X-ray powder diffraction patterns, (**b**) FTIR spectra, and (**c**,**d**) optical properties analysis: (**c**) UV-Vis absorption spectra and (**d**) calculated bandgap values of as-prepared Co_2_SnO_4_, WS_2_, NHCSs, Co_2_SnO_4_/WS_2_, Co_2_SnO_4_@NHCSs, WS_2_@NHCSs, and Co_2_SnO_4_/WS_2_@NHCSs heterostructures.

The X-ray diffraction (XRD) technique was utilized to analyze the crystalline structure of the samples. X-ray diffraction (XRD) findings were obtained from a variety of photocatalysts, including Co_2_SnO_4_, WS_2_, NHCSs, Co_2_SnO_4_/WS_2_, Co_2_SnO_4_@NHCSs, WS_2_@NHCSs, and Co_2_SnO_4_/WS_2_@NHCSs heterostructures. These results are illustrated in Figure 1a. At 2θ values of 17.76°, 29.08°, 34.39°, 35.91°, 41.75°, 51.78°, 55.18°, 60.55°, 63.57°, 71.41°, 72.40°, and 78.08°, the diffraction peaks of Co_2_SnO_4_ nanocubes were measured and observed. There is a correlation between these peaks and the crystal planes of (111), (220), (311), (222), (400), (422), (511), (440), (531), (533), (622), and (551), respectively. The diffraction pattern that was discovered is in agreement with the conventional cubic Co_2_SnO_4_ (JCPDS No. 029–0514) [40]. The XRD pattern of WS_2_ nanosheets exhibited pronounced diffraction peaks at 14.97°, 30.08°, 45.81°, and 59.34°, corresponding to the (002), (004), (006), and (008) planes of WS_2_, respectively. The prominent peak at 14.97° signifies the systematic arrangement of S-W-S strata [41]. All of them are consistent with those documented for WS_2_ 3D nanoflowers in standard JCPDS card No. 00-08-0237 [42]. The distinct profiles of the reflections indicate excellent crystallization of the material. Figure 1a illustrates the X-ray diffraction patterns of NHCSs and their nanocomposites. The prominent peak at 24° was ascribed to the amorphous carbon present in the NHCSs [36]. The XRD investigation of Co_2_SnO_4_/WS_2_, Co_2_SnO_4_@NHCSs, WS_2_@NHCSs, and Co_2_SnO_4_/WS_2_@NHCSs heterostructures revealed no peaks attributable to impurities, indicating the complete production of nanostructures.

The average crystallite size (D) of the catalysts was determined from the most prominent diffraction peak utilizing Scherrer’s formula.

D = kλcosθ(1)

where D refers to the particle size, refers to peak width at half maximum intensity (FWHM) in radians, λ refers to the X-ray wavelength, and θ is the Bragg angle corresponding to the diffraction peak.

Figure 1b illustrates the FT-IR spectroscopy properties of bare WS_2_, Co_2_SnO_4_, NHCSs, and their respective nanocomposite materials, including Co_2_SnO_4_/WS_2_, Co_2_SnO_4_@NHCSs, WS_2_@NHCSs, and Co_2_SnO_4_/WS_2_@NHCSs heterostructures. Figure 1b indicates that the band at 634 cm^−1^ and 892 cm^−1^ corresponds to the W–S bond, whereas the band at 1083 cm^−1^ is likely associated with S–S bonds [43]. The peaks at 1392 and 1625 cm^−1^ signify hydroxyl groups [44,45] inside the WS_2_ framework. The vibrational bands seen at around 3440 cm^−1^ are attributed to ambient OH resulting from moisture adsorption on the surface of WS_2_ [46,47]. Furthermore, the two prominent absorption bands at 572 and 641 cm^−1^ correspond to the Co–O and Sn–O vibrations, respectively. The absorption peak identified at a wavenumber of 418–620 cm^−1^ is attributed to the stretching vibration of the Co-O^2+^ and Co-O^3+^ bonds [48]. The two weak absorptions at 1641 and 3429 cm^−1^ correspond to the bending vibration of H–O–H and the symmetric stretching vibration of OH in lattice water. The FTIR spectra of the NHCSs sample display distinctive absorption peaks at 3448 cm^−1^, signifying O–H stretching vibrations [47]. The peaks detected at 2947 cm^−1^ correspond to C–H stretching vibrations. The peak at 1621 cm^−1^ corresponds to C–C/C=C stretching vibrations, whereas the peak at 1392 cm^−1^ is linked to OH bending vibrations. The peak at 1237 cm^−1^ corresponds to C–O stretching vibrations. Moreover, the peak at 1112 cm^−1^ signifies C–N stretching vibrations. These peaks collectively indicate that NHCSs consist of diverse functional groupings [49]. Figure 1b illustrates the FTIR spectrum of the Co_2_SnO_4_/WS_2_, Co_2_SnO_4_@NHCSs, WS_2_@NHCSs, and Co_2_SnO_4_/WS_2_@NHCSs heterostructures. The characteristic peaks of bare Co_2_SnO_4_ and WS_2_ shift towards lower wavenumbers following the integration of NHCSs, indicating an interaction among Co_2_SnO_4_, WS_2_, and NHCSs. Nonetheless, numerous peaks within the range of 1000 to 1600 cm^−1^ are challenging to differentiate on the nanocomposite curve owing to the minimal presence of NHCSs. The spectra exhibited all characteristic peaks of Co_2_SnO_4_ and WS_2_ in the Co_2_SnO_4_/WS_2_@NHCSs nanocomposite. Consequently, FTIR analysis validated the successful synthesis of the nanocomposite, consistent with the XPS spectra.

### 2.2. Optical Properties Analysis

As shown in Figure 1c,d, the optical properties of the collected heterostructures (Co_2_SnO_4_, WS_2_, NHCSs, Co_2_SnO_4_/WS_2_, Co_2_SnO_4_@NHCSs, WS_2_@NHCSs, and Co_2_SnO_4_/WS_2_@NHCSs) are examined using UV-Vis DRS absorption spectra spanning 200 to 800 nm. The pure Co_2_SnO_4_, WS_2_, and NHCSs display a broad visible-light absorption (~620 nm) characteristic, with an absorption edge at around 600 nm that is consistent with a previously recorded result. The development of heterostructure also accounts for the broader responses when contrasted with others. The following equation calculates the bandgap energy value of the Co_2_SnO_4_/WS_2_@NHCSs heterostructures: using this equation, the as-obtained samples’ optical bandgap energy (Eg) has been determined:

αhv = A (hν − Eg)^n/2^ (2)

where h, α, ν, and A represent the Planck constant, absorption coefficient, light frequency, and proportional constant, respectively. According to Figure 1c,d, the optical Eg values of the following heterostructures are calculated to be 1.63 eV [26], 1.86 eV [50], 1.56 eV, 1.52 eV, 1.71 eV, 1.46 eV, and 1.43 eV for Co_2_SnO_4_, WS_2_, NHCSs, Co_2_SnO_4_/WS_2_, Co_2_SnO_4_@NHCSs, WS_2_@NHCSs, and Co_2_SnO_4_/WS_2_@NHCSs, respectively. A combination of the ideal low bandgap of WS_2_ and CO_2_SnO_4_ with the NHCSs content in the Co_2_SnO_4_/WS_2_@NHCSs heterostructures surface, along with strong interfacial coupling effects, may explain the decreased bandgap values of these heterostructures. This, in turn, could lead to improved photocatalytic action and greater visible-light utilization.

### 2.3. Morphology Structure Analysis

**Figure 2:**
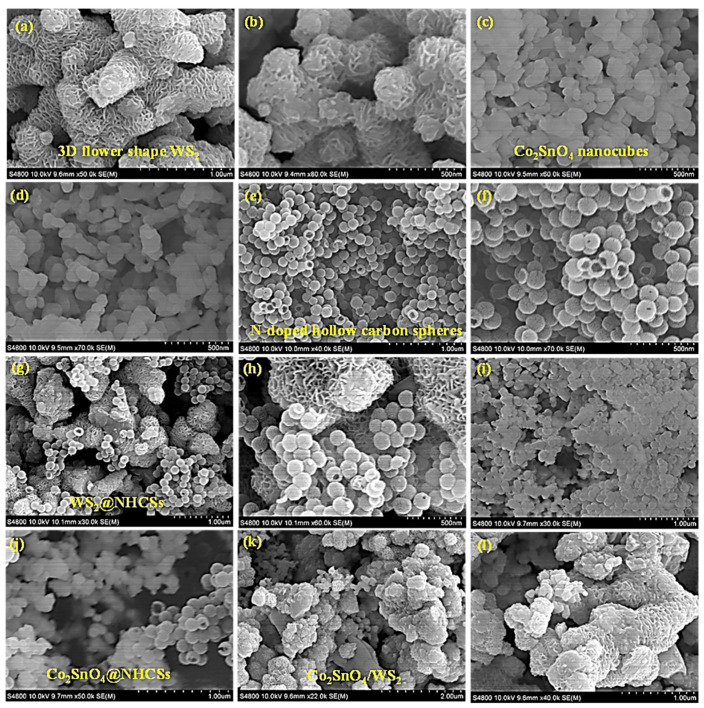
FE-SEM images of (**a**,**b**) WS_2_, (**c**,**d**) Co_2_SnO_4_, (**e**,**f**) NHCSs, (**g**,**h**) WS_2_@NHCSs, (**i**,**j**) Co_2_SnO_4_@NHCSs, and (**k**,**l**) Co_2_SnO_4_/WS_2_ heterostructures with different magnification ranges.

The materials produced by the hydrothermal reaction and the high-temperature solid-state reaction are shown in Figure 2 by utilizing scanning electron microscopy. The observed size of the nanocubes is around 100 nm, and Co_2_SnO_4_ appears to have an ordered cubical shape with interconnected nano crystallites (Figure 2c,d). To deduce the nanoflower structure of WS_2_, scanning electron microscopy (SEM) pictures of as-synthesized 3D hierarchical nanoflowers were captured at various magnifications and are displayed in Figure 2a,b. On average, WS_2_ nanosheets with a diameter of ~17 nm contain nanoscale patchy particles that have both thick and thin nanosheets.

**Figure 3:**
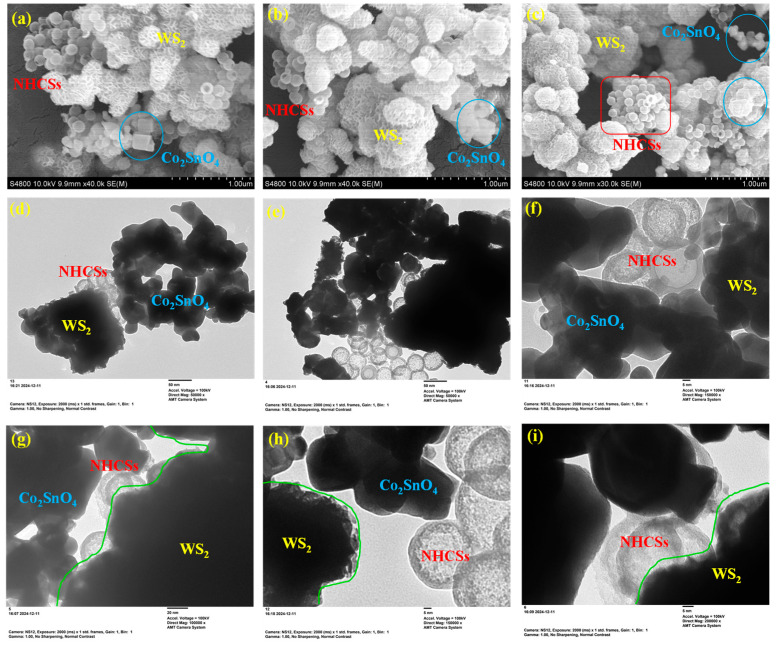
Morphological analysis: (**a**–**c**) FE-SEM images of Co_2_SnO_4_/WS_2_@NHCSs heterostructures and (**d**–**i**) HR-TEM images of (**a**–**e**) Co_2_SnO_4_/WS_2_@NHCSs heterostructures with different magnification ranges.

The SEM image, captured at high magnification, showed revealed that nanoparticles in the sample were loosely stacked. The nanoparticles have a width ranging from 100 to 200 nm and a thickness of approximately 10 nm. The NHCSs have a consistent and spherical shape with an average diameter of around 150—160 nm, as seen in the scanning electron micrograph (SEM) image in Figure 2e,f. The SEM images in Figure 2g,h represent the WS_2_@NHCSs, Figure 2i,j represents the Co_2_SnO_4_@NHCSs, and Figure 2k,l represents the Co_2_SnO_4_/WS_2_ heterostructures. From the results, the host materials like Co_2_SnO_4_ and WS_2_ are well dispersed with the NHCSs. From Figure 3a–c, it shows the Co_2_SnO_4_/WS_2_@NHCSs heterostructures, in this structures Co_2_SnO_4_ nanocubes and the NHCSs nanospheres are well dispersed with the surface of WS_2_ nanoflowers. This will be confirmed the formation of the Co_2_SnO_4_/WS_2_@NHCSs heterostructure. The production of nanocube, 3D nanoflower, and nanosphere structures is clearly indicated by the HRTEM picture of Co_2_SnO_4_/WS_2_@NHCSs (Figure 3d–i). The findings show that Co_2_SnO_4_ was effectively covered with the surface of WS_2_ nanoflowers and was well agglomerated, as shown in the high-resolution transmission electron microscopy (HR-TEM) images. The transmission electron micrographs of the NHCSs show an internal structure with a black outside and a light inside. It was found that the shell had a thickness of around 50 nm.

### 2.4. Elemental Composition Analysis

**Figure 4:**
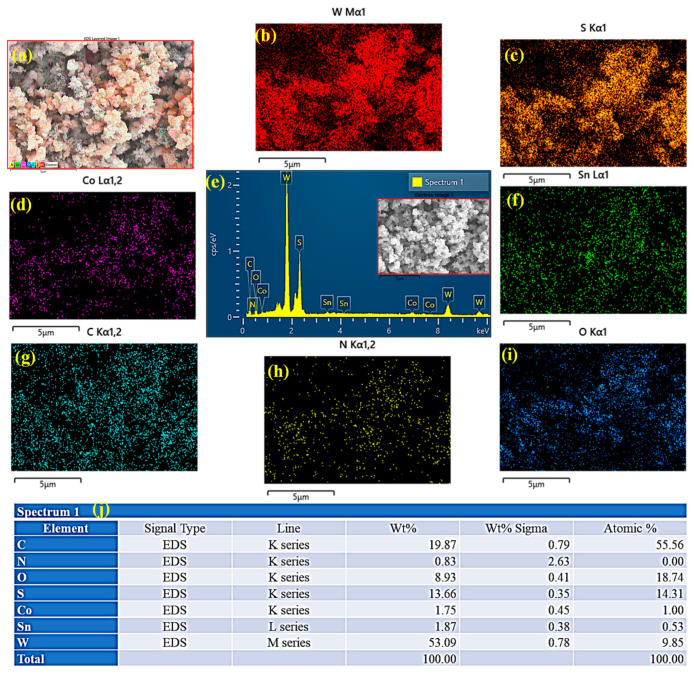
Elemental composition analysis. (**a**) Elemental mapping overlay of Co_2_SnO_4_/WS_2_@NHCSs heterostructures, (**b**) the corresponding mapping images of tungsten, (**c**) sulfur, (**d**) cobalt, (**e**) EDX spectra the Co_2_SnO_4_/WS_2_@NHCSs heterostructures, (inset) the corresponding SEM image of the selected area for elemental mapping, (**f**) tin, (**g**) carbon, (**h**) nitrogen, (**i**) oxygen, and (**j**) table of atomic percentage of atoms present in the Co_2_SnO_4_/WS_2_@NHCSs heterostructures.

EDX analysis confirmed the presence of Co_2_SnO_4_, WS_2_, and NHCSs in the Co_2_SnO_4_/WS_2_@NHCSs heterostructure. Figure 4a,b illustrates the associated SEM areal picture and EDX spectrum of a Co_2_SnO_4_/WS_2_@NHCSs heterostructure photocatalyst. The results indicate that the composite material exhibits unique peaks for the elements Co, Sn, O, W, S, C, and N, with no further identifiable peaks present (Figure 4c). This denotes the examination of the Co_2_SnO_4_/WS_2_@NHCSs heterostructure composition via EDX analysis. The Co_2_SnO_4_/WS_2_@NHCSs heterostructure consisted of Co (1.75%), Sn (1.87%), O (8.93%), W (56.71%), S (13.66%), C (19.87%), and N (0.83%). The results obtained confirm the successful synthesis of the Co_2_SnO_4_/WS_2_@NHCSs heterostructure photocatalyst. The heterostructure demonstrates an absence of contaminants, hence validating the findings from the powder XRD. The EDX results indicate that the mapping examination of the nanocomposite, depicted in Figure 4d–i, validates the uniform distribution of Co, Sn, O, W, S, C, and N elements throughout the sample.

### 2.5. XPS Analysis

**Figure 5:**
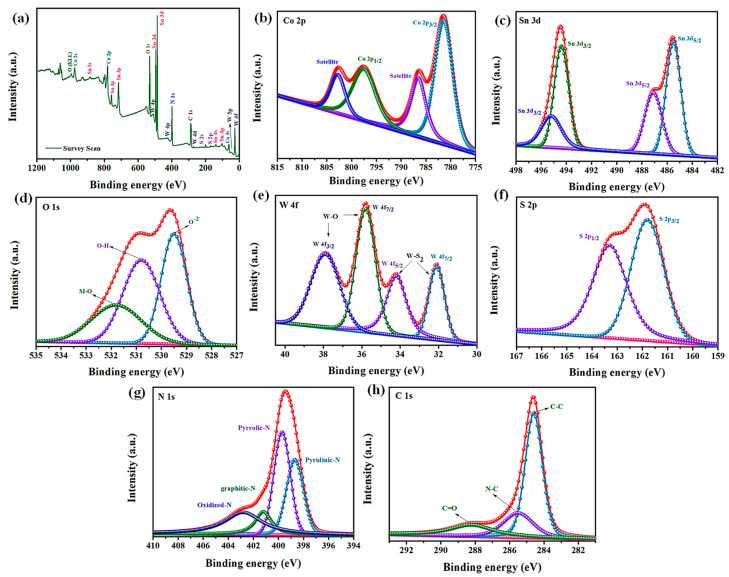
Elemental composition analysis: XPS spectrum of as-obtained Co_2_SnO_4_/WS_2_@NHCSs nanocomposite, (**a**) survey spectrum, and (**b**–**h**) high-resolution images of the individual elements, (**b**) Co 2p, (**c**) Sn 3d, (**d**) O 1 s, (**e**) W4f, (**f**) S 2p, (**g**) N 1 s, and (**h**) C 1 s elements.

The XPS survey spectrum of the Co_2_SnO_4_/WS_2_@NHCSs nanocomposite, depicted in Figure 5, indicated the existence of the elements Co, Sn, W, S, C, O, and N within the heterostructure. The survey scan of the Co_2_SnO_4_/WS_2_@NHCSs nanocomposite is depicted in Figure 5a. As shown in Figure 5b, the XPS analysis produced a complete spectrum of Co 2p. The coefficients of 2p_3/2_ and 2p_1/2_, which are located at approximately 781.5 and 797.6 eV, respectively, are the two detected peaks. According to Reference [51], the 786.6 and 802.9 eV satellite peaks are typically caused by multi-electron excitation or the interaction of unpaired electrons. Sn 3d_5/2_ is assigned the major peak with binding energies of 485.4 eV and 487.1 eV in the X-ray photoelectron spectroscopy (XPS) spectrum of Sn 3d, as shown in Figure 5c. Sn 3d_3/2_ is assigned the peaks at 494.4 eV and 495.3 eV [52]. Figure 5d presents the high-resolution O 1s spectra of Co_2_SnO_4_/WS_2_@NHCSs. The O 1s XPS spectrum exhibits three peaks at 529.5 eV, 530.8 eV, and 531.8 eV, corresponding to O^−2^, metal-oxygen, and O–H bonds on the catalyst surface, respectively, indicating the existence of divalent oxygen in the catalyst [53]. The high-resolution W 4f spectra (Figure 5e) of WS_2_ displayed binding energies of 32.1 eV and 34.2 eV, corresponding to W 4f_7/2_ and W 4f_5/2_, respectively [54]. In the Co_2_SnO_4_/WS_2_@NHCSs heterostructure, the binding energies of 32.0 eV and 34.1 eV were attributed to the W 4f_7/2_ and W 4f_5/2_ characteristic peaks of WS_2_. The binding energies of 35.7 eV and 37.9 eV correspond to the typical peaks of the W-O bond [55]. The core-level S 2p spectra (Figure 5f) exhibited two distinct peaks at 163.3 eV and 161.8 eV, corresponding to S 2p_1/2_ and S 2p_3/2_, respectively [56]. In Figure 5g, the C 1s XPS spectra exhibit three deconvoluted peaks located at 284.7 eV (indicative of the sp^2^ C–C bond), 285.5 eV (N–C bond), and 288.3 eV (C–O bond). The deconvoluted N 1s XPS spectra of the Co_2_SnO_4_/WS_2_@NHCSs sample reveal four peaks: pyridinic N at 398.7 eV, pyrrolic N at 399.7 eV, graphitic N at 401.2 eV, and oxidized N at 402.9 eV, respectively [38]. Integrating nitrogen atoms into carbon materials can improve their surface area, porosity, conductivity, and stability. These enhancements are essential for augmenting their degradation efficacy in photocatalytic applications. The inclusion of all constituents validates the effective synthesis of Co_2_SnO_4_/WS_2_@NHCSs nanocomposites.

### 2.6. Photocatalytic Degradation Performance of CR Dye

**Figure 6:**
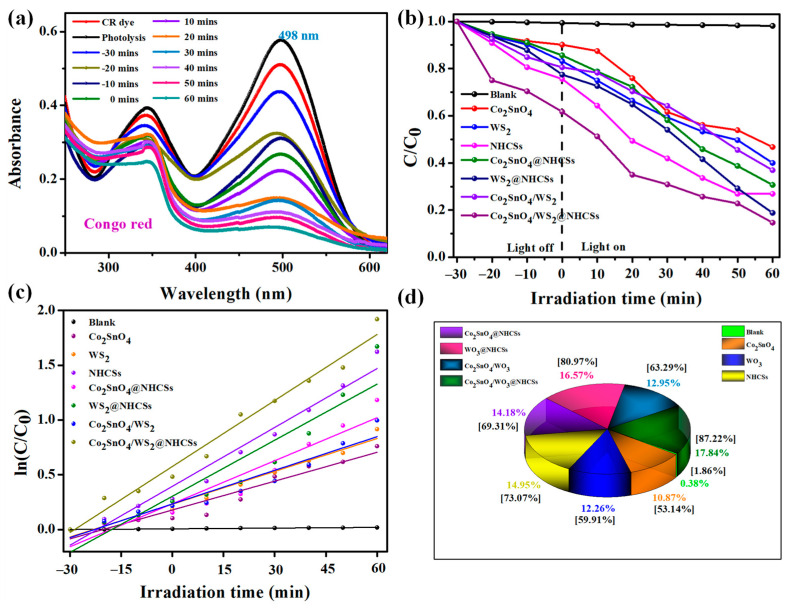
Photodegradation of CR dye under various optimized conditions: (**a**) Absorbance spectrum of CR dye degradation using Co_2_SnO_4_/WS_2_@NHCSs heterostructures, (**b**) different catalyst usage (catalysts: 30 mg/L, CR conc.: 5 mg/L), (**c**) First-order kinetics model of CR dye degradation. (**d**) Degradation efficiency of different catalysts usage.

The degradation of aqueous CR dye under visible-light exposure was another way the photocatalytic degradation performances of the heterostructure photocatalysts (PCs) of Co_2_SnO_4_/WS_2_@NHCSs were evaluated (Figure 6a). The concentration ratio before and after a certain reaction time, as shown by the C/C_0_ plot, was used to define the photodegradation efficiency of PCs. During the adsorption-desorption time, all of the PCs that were obtained had a low adsorption for mixed dye molecules, less than 2%. The stability of the mixed pollutant under these exact reaction conditions was established when no detectable photodegradation was observed in the blank (self-degradation) test without the presence of catalyst. Thus, without photocatalysts (PCs), the photolysis of CR dye molecules is extremely low, reaching only 1.86% in 60 min. The degradation process revealed that after 60 min of light exposure, the following CR dye decomposition efficiencies were observed over the various PCs: as-obtained Co_2_SnO_4_, WS_2_, NHCSs, Co_2_SnO_4_/WS_2_, Co_2_SnO_4_@NHCSs, WS_2_@NHCSs, and Co_2_SnO_4_/WS_2_@NHCSs had values of 53.14%, 59.91%, 73.07%, 63.29%, 69.31%, 80.97%, and 87.22%, respectively (Figure 6d). In addition, the heterojunction catalysts made of as-obtained Co_2_SnO_4_ and WS_2_@NHCSs demonstrate better photocatalytic activities for the degradation of CR dyes when exposed to visible light for 60 min, compared to an aqueous dye removal efficiency of 87.22% (Figure 6b).

Figure 6c illustrates a linear correlation between –ln(C_t_/C_0_) and kt, which was plotted and fitted to the pseudo-first-order kinetic model. In this context, k, C_0_, and C_t_ represent the pseudo-first-order rate constant (min^−1^), the initial concentration of the assumed dye, and the concentration at a subsequent time interval during the catalytic reaction at time t [16]. The k values for the Co_2_SnO_4_, WS_2_, NHCSs, Co_2_SnO_4_/WS_2_, Co_2_SnO_4_@NHCSs, WS_2_@NHCSs, and Co_2_SnO_4_/WS_2_@NHCSs photocatalysts in the CR dye decomposition reaction were calculated as 0.0087 min^−1^, 0.0099 min^−1^, 0.0179 min^−1^, 0.0131 min^−1^, 0.0171 min^−1^, 0.0103 min^−1^, and 0.0201 min^−1^, respectively, indicating that the Co_2_SnO_4_/WS_2_@NHCSs photocatalyst exhibits the highest value among the catalysts tested. Table 1 presents the photocatalytic degradation efficiency, apparent reaction rate constant (k), and R² values of the obtained catalyst samples.

**Figure 7:**
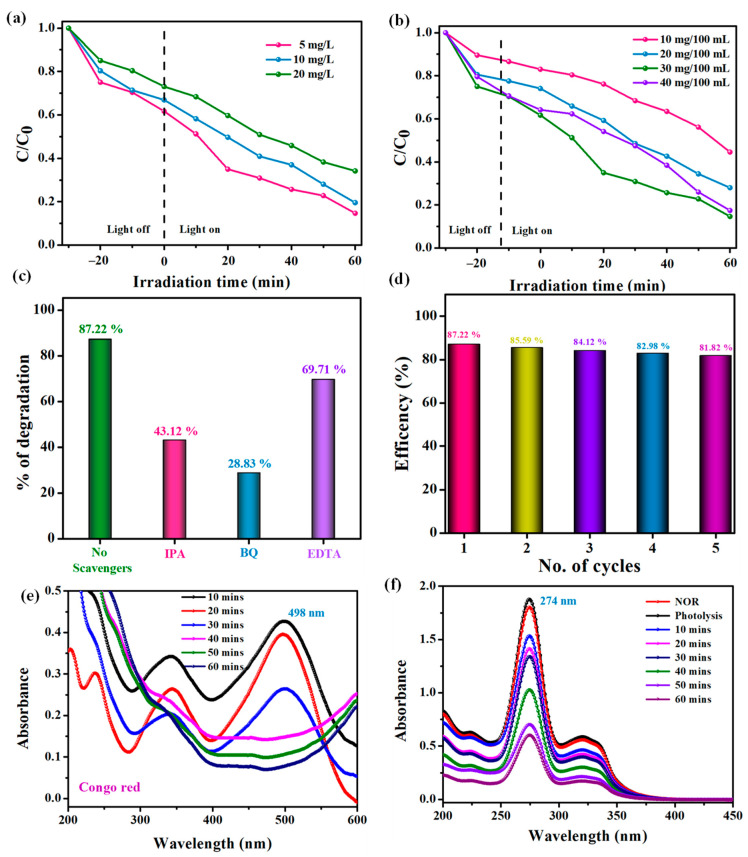
Photodegradation of CR dye under various optimized conditions. (**a**) Optimization of different catalyst usage (CR conc.: 5 mg/L). (**b**) Optimization of CR dye concentration (catalysts: 30 mg/L). (**c**) Influence of different radical ions on the visible-light-induced degradation of CR dye. (**d**) Reusability and stability analysis of Co_2_SnO_4_/WS_2_@NHCSs heterostructures for CR degradation. (**e**) Absorbance spectrum of CR dye degradation after the fifth cycle. (**f**) Absorbance spectrum of the antibiotic norfloxacin (NOR)’s degradation under visible-light irradiation.

The influence of different initial dye concentrations on the degradation efficiency of CR dye is evaluated by altering the concentration range from 5 mg/L to 20 mg/L under visible-light exposure. The quantity of photocatalyst employed for CR degradation was consistently set at 30 mg. The photocatalytic outcomes are illustrated in Figure 7a. The findings indicate that the photocatalytic efficiency of the Co_2_SnO_4_/WS_2_@NHCSs photocatalysts is inversely related to the dye concentration under the same conditions. The highest degradation efficiency was observed at the minimal dye concentration of 5 mg/L. The degradation of CR diminished progressively from approximately 87.22% to about 65.82% as the dye concentration increased from 5 mg/L to 20 mg/L. The diminished light absorption on the photocatalyst’s surface results from the increased dye concentration. The research indicates that the catalyst exhibits optimal degradation efficiency at a dye concentration of 5 mg/L. Consequently, it can be deduced that the photocatalyst is optimally utilized for the degradation of CR dye at low concentration levels.

A varying quantity of photocatalysts (10 mg/100 mL to 40 mg/100 mL) was employed to adjust the catalyst dosage (Figure 7b). The findings indicated that the optimal degradation efficiency for Co_2_SnO_4_/WS_2_@NHCSs photocatalysts was achieved with 30 mg of photocatalyst per 100 mL. Nonetheless, despite the increased quantity of photocatalyst, the degradation efficiency remained significantly inferior to that achieved with Co_2_SnO_4_/WS_2_@NHCSs photocatalysts. This emphasized the crucial influence of the catalyst support on degradation efficiency. The deterioration was markedly reduced when the catalyst dose exceeded the optimal levels, since the incident radiation was largely dispersed by the aggregated photocatalyst. The impact of catalyst concentration on degradation efficiency was also examined. The increase in catalyst content resulted in a reduction in degradation efficiency due to the availability of additional active sites.

Radical trapping experiments were conducted to elucidate the primary active radicals generated by the Co_2_SnO_4_/WS_2_@NHCSs ternary heterostructures during the photocatalytic degradation of CR dye. In this context, p-benzoquinone (BQ) serves as a quencher for the superoxide radical (^•^O_2_^−^), whereas ethylenediaminetetraacetate disodium (EDTA-2Na) and isopropyl alcohol (IPA) function as quenchers for holes (h^+^) and hydroxyl radicals (^•^OH), respectively (Figure 7c). The use of EDTA-2Na as a proton scavenger had a minimal impact on the degradation efficiency of CR dye, suggesting that the involvement of protons is nearly insignificant in the reaction system. The addition of IPA significantly inhibited the CR dye removal rate, underscoring the critical role of ^•^OH in the catalytic reaction. The addition of BQ markedly decreased photocatalytic performance, indicating that ^•^O_2_^−^ is crucial for CR breakdown. The study determined that the CR dye molecule was eliminated more swiftly with the aid of ^•^O_2_^−^ and ^•^OH radicals while utilizing the Co_2_SnO_4_/WS_2_@NHCSs catalyst.

The photo-stability of Co_2_SnO_4_/WS_2_@NHCSs PCs is further examined in the recycling tests because of the practical importance of the PCs’ recyclability and durability in their applied uses. After five consecutive recycles, the good photodegradation rate (87.22–81.82%) for the CR dye aqueous solution is still maintained by the Co_2_SnO_4_/WS_2_@NHCSs ternary PCs, as shown in Figure 7d. The catalyst is lost during the washing and drying process, which is responsible for the little decrease in photocatalytic activity (< 6%) after five consecutive cycles [57]. This means that the Co_2_SnO_4_/WS_2_@NHCSs PCs can be easily recycled and reused without suffering a major degradation in the photo-reaction process, even after five repetitions [58]. Figure 7e shows the absorbance spectra of the CR dye after 60 min of irradiation, demonstrating the improved degradation efficiency throughout five cycles. Table 2 represents the comparison study of WS_2_ based photocatalyst for the degradation of organic pollutants [26,44,59,60,61,62]. In addition, the heterojunction catalysts made of as-obtained Co_2_SnO_4_/WS_2_@NHCSs demonstrate better photocatalytic activities for the degradation of the antibiotic norfloxacin (NOR) under visible-light irradiation. The Co_2_SnO_4_/WS_2_@NHCSs nanocomposites has achieved the degradation efficiency of 68.7% within 60 min (Figure 7f).

**Figure 8:**
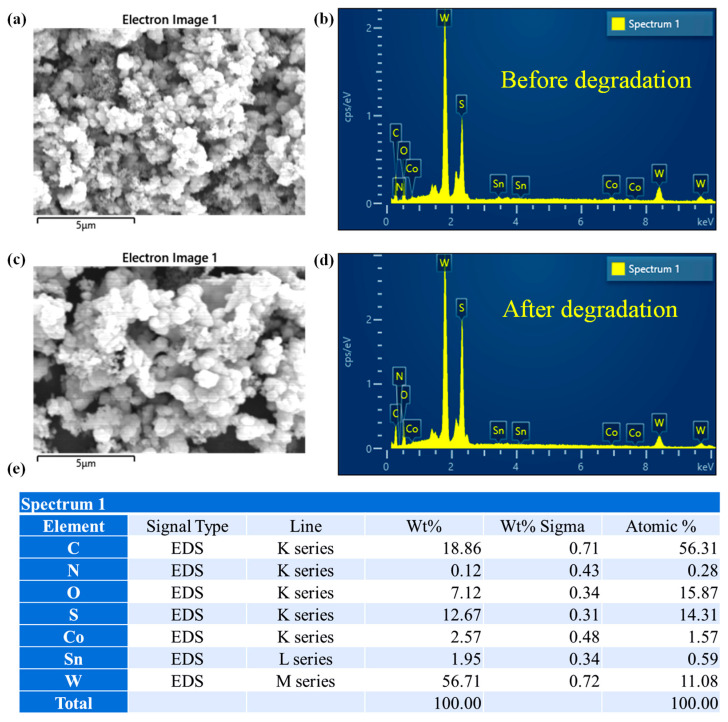
Elemental composition analysis (**a**,**b**) corresponding SEM image and EDX analysis of Co_2_SnO_4_/WS_2_@NHCSs heterostructures before CR dye degradation and (**c**,**d**) corresponding SEM images and EDX spectra of the Co_2_SnO_4_/WS_2_@NHCSs heterostructures after CR dye degradation. (**e**) The quantitative results of weight and atomic concentration of the selected elements on the basis of EDS analysis of Co_2_SnO_4_/WS_2_@NHCSs heterostructures after CR dye degradation.

After the recycling analysis, to investigate the elemental composition, the elemental compositions of the Co_2_SnO_4_/WS_2_@NHCSs heterostructures were studied before and after the CR dye degradation (Figure 8). After the degradation process, the results from the EDX examination showed that all components were present without any contaminants (Figure 8d). After five cycles, the results showed that the photocatalyst fabricated from Co_2_SnO_4_/WS_2_@NHCSs was more stable and had a better degradation ability.

### 2.7. Mott–Schottky Plot Analysis

**Figure 9:**
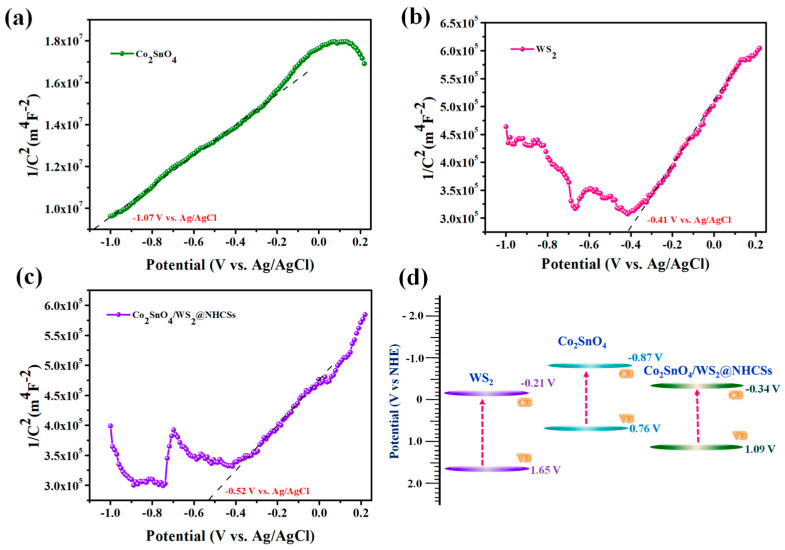
Mott–Schottky plots of bare (**a**) Co_2_SnO_4_, (**b**) WS_2_, and (**c**) Co_2_SnO_4_/WS_2_@NHCSs nanocomposites (1500 Hz) in 0.1 M KOH aqueous solution and (**d**) band energy diagram of the various nano structures.

Mott–Schottky measurement was conducted to ascertain the flat band potential (E_fb_) of bare Co_2_SnO_4_, WS_2_, and Co_2_SnO_4_/WS_2_@NHCSs nanocomposites (at 1500 Hz), as seen in Figure 9a–c, to elucidate the photodegradation mechanism. The flat band potential values of bare Co_2_SnO_4_, WS_2_, and Co_2_SnO_4_/WS_2_@NHCSs nanocomposites were −1.07 V, −0.41 V, and −0.54 V, respectively, relative to the Ag/AgCl electrode. The negative slopes of the curves for bare Co_2_SnO_4_, WS_2_, and Co_2_SnO_4_/WS_2_@NHCSs nanocomposites indicate their p-type semiconductor characteristics, finally forming a p-p type heterojunction as described in the literature [63]. Utilizing the equation ENHE = Ag/AgCl + 0.197, the newly determined conduction band values at NHE for bare Co_2_SnO_4_, WS_2_, and Co_2_SnO_4_/WS_2_@NHCSs nanocomposites are −0.873 V, −0.213 V, and −0.343 V, respectively, as derived from DRS-UV findings. The conduction band of the semiconductor is often equivalent to the flat band potential. The conduction band positions of bare Co_2_SnO_4_, WS_2_, and Co_2_SnO_4_/WS_2_@NHCSs nanocomposites were around −0.873 V, −0.213 V, and −0.343 V, respectively. The valence band (VB) of bare Co_2_SnO_4_, WS_2_, and Co_2_SnO_4_/WS_2_@NHCSs nanocomposites can be quantified using the specified equation [64].

E_VB_ = E_CB_ + Eg (3)

where Eg represents the energy value of the bandgap. Consequently, it can be inferred that the valence band energies (E_VB_) are 1.65 V, 0.75 V, and 1.09 V (against NHE) for bare Co_2_SnO_4_, WS_2_, and Co_2_SnO_4_/WS_2_@NHCSs nanocomposites, respectively.

### 2.8. Photocatalytic Degradation Mechanism

**Scheme 1:**
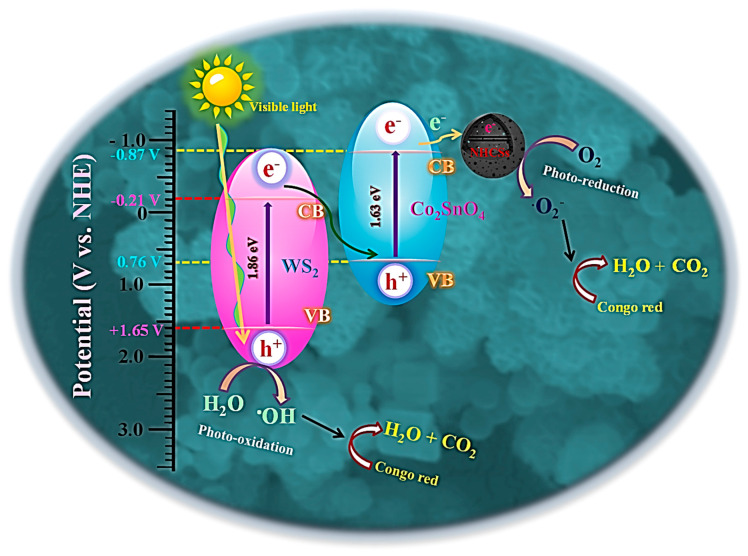
Schematic diagram of the proposed degradation mechanism for the photocatalytic degradation of CR dye using Co_2_SnO_4_/WS_2_@NHCSs heterostructures.

Based on the aforementioned experimental results, a plausible charge-carrier transport mechanism for the improved photocatalytic activity of Co_2_SnO_4_/WS_2_@NHCSs heterojunctions has been proposed, as illustrated in Scheme 1. Upon exposure to visible light, both Co_2_SnO_4_ and WS_2_ were able to undergo photoexcitation, resulting in the generation of electron–hole pairs in their respective valence (VB) and conduction bands (CB). The photogenerated holes (h^+^) and electrons (e^−^) combine with hydroxyl ions (OH^−^) and oxygen (O_2_) to produce reactive oxygen species such as hydroxyl radicals (OH^•^) and superoxide radical anions (^•^O_2_^–^). The reactive oxygen species on the photocatalyst’s surface facilitate the efficient photodegradation of contaminants. Mott–Schottky analysis (Figure 9) yielded the calculated conduction band values for Co_2_SnO_4_ and WS_2_. The conduction band (CB) values of Co_2_SnO_4_ and WS_2_ are −0.87 V and −0.21 V, respectively. The bandgap energies of Co_2_SnO_4_ and WS_2_ are 1.86 eV and 1.63 eV, respectively, as determined from the K-M function derived from UV-DRS spectra (Figure 1d). Additionally, the energy of the valence band (VB) was determined using the equation E_VB_ = E_CB_ + Eg, where E_VB_ represents the VB value, E_CB_ denotes the CB value, and Eg signifies the bandgap energy of the catalysts [65]. According to the aforementioned formula, the CB values for Co_2_SnO_4_ and WS_2_ were 0.76 V and 1.65 V, respectively. Consequently, photoinduced holes (h^+^) in the valence band of WS_2_ facilitated the generation of hydroxyl radicals (^•^OH) from hydroxyl ions (OH^−^). The Z-scheme process indicates that photoinduced electrons transition from the conduction band of WS_2_ to recombine with holes in the valence band of Co_2_SnO_4_, hence enhancing the separation of photogenerated electron–hole pairs. The transferred electrons subsequently migrate to adjacent NHCSs from the conduction band of Co_2_SnO_4_. As electrons amassed on the surface of the CSs, dissolved O_2_ (h^+^) was reduced by these electrons to yield ^•^O_2_^–^, subsequently leading to the generation of ^•^OH by the reaction of ^•^O_2_^–^ with electrons, while the vacancies in the VB of WS_2_ may directly engage with organic contaminants. As a result, the synergistic catalytic effects achieved through interfacial manipulation and the construction of charge-transfer routes among the three components enhanced photodegradation efficiency.

### 2.9. Photocatalytic Degradation Pathway Analysis

**Figure 10:**
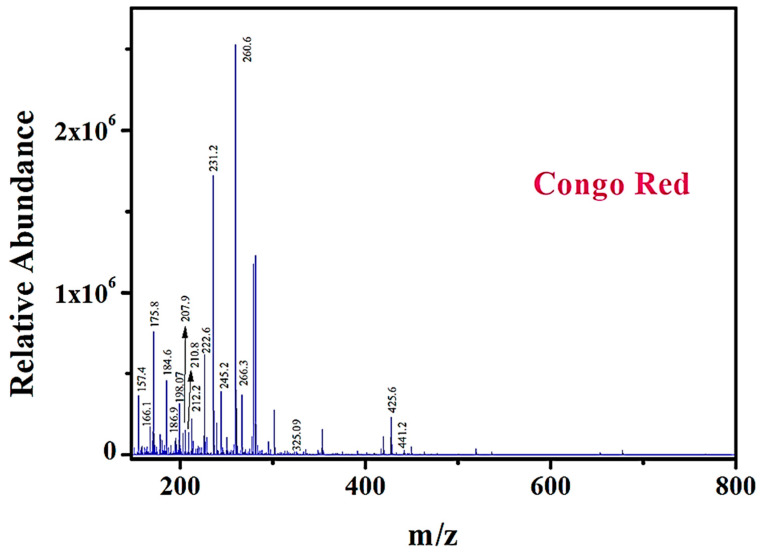
Corresponding LC-MS spectra for the photodegradation products of CR dye using Co_2_SnO_4_/WS_2_@NHCSs heterostructures.

**Scheme 2:**
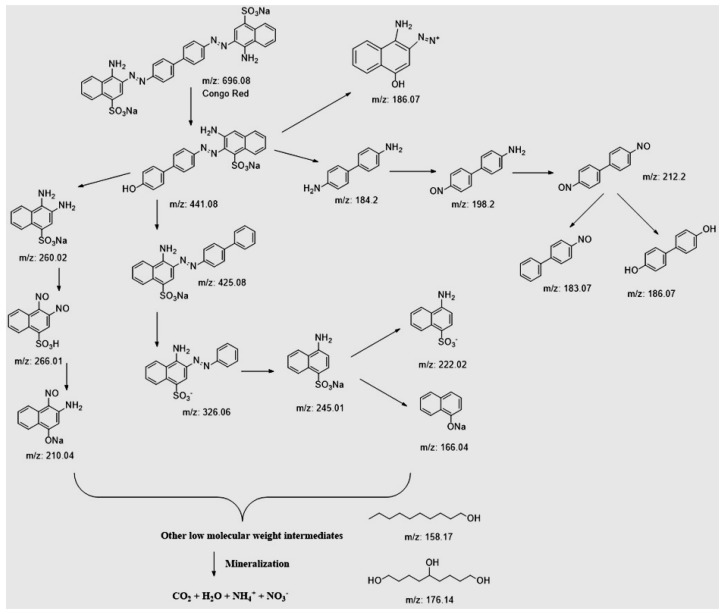
The proposed degradation pathway of CR dye using Co_2_SnO_4_/WS_2_@NHCSs heterostructures.

An exhaustive investigation of the degradation process and potential reaction intermediates enabled us to ascertain the degradation mechanism. The photodegraded CR dye solution was examined utilizing LC-MS (Figure 10). Significant intermediate species were identified in the CR solution during the degradation process utilizing the Co_2_SnO_4_/WS_2_@NHCSs heterojunction catalyst (30 mg/L), as per the LC-MS analysis. Scheme 2 illustrates the species identified during various temporal intervals. The subsequent stages result in the breakdown of CR dye: A direct dissociation of the benzene ring transpires. The aromatic ring and sulfonate groups no longer establish a –C–S–bond. Numerous chromophore groups, specifically –C–N– and –C–C– bonds, are cleaved. The electron–hole pair (–N=N–) is dissociated [66]. The Congo red molecule exhibits fragmentation with several moieties at distinct m/z values. The intermediates may be generated through desulfonation, hydroxylation for deamination, nitration, and subsequent hydroxylation for further deamination, yielding m/z values of 441, 425, 326, 266, 260, 245, 198, 184, 212, 210, and 166, which can be made by cleaving the –N=N– bond. Dye molecules are simplified to their metabolites through deamination, amine oxidation, and desulfonation, resulting in the formation of intermediates. Scheme 2 verifies the potential degradation and the resultant fragments of CR dye during the degradation process.

## 3. Materials and Methods

### 3.1. Material Usage

In this work, the required chemicals used for the synthesis of Co_2_SnO_4_/WS_2_@NHCSs heterostructures, including CoCl_2_·H_2_O (99%), SnCl_4_·5H_2_O (98%), methanol (CH_3_OH, ~99.99%), urea, melamine (99%), Sodium tungstate dihydrate (Na_2_WO_4_·2H_2_O, 96%), and thiourea (CH_4_N_2_S) were purchased from Daejung chemicals (Siheung, South Korea). Tetraethyl orthosilicate (TEOS, 99%), resorcinol (99%), hydrofluoric acid (HF, ≥48% in water), formaldehyde (a 37 wt% aqueous solution stabilized with 5–15% methanol), Cetyltrimethylammonium bromide (CTAB, 99%), and absolute ethanol (99.8%) were purchased from Sigma-Aldrich (MERK, Darmstadt, Germany). Aqueous ammonia solution (25.0–30.0%) was purchased from Samchun Pure Chemical Co. (Seoul, South Korea). All chemicals were used as received without further purification.

### 3.2. Synthesis of Co_2_SnO_4_

To create transparent solutions, CoCl_2_·6H_2_O and SnCl_4_·5H_2_O were dissolved in distilled water and vigorously stirred at 450 rpm. The two solutions were mixed, then NaOH solutions were magnetically swirled into the Co-Sn solution. This method mineralized using NaOH. Addition of urea aqueous solution occurred simultaneously. After the chemical reaction, a greenish tin and cobalt hydroxide suspension solution was obtained. Afterward, the mixture was placed in a PTFE-lined stainless autoclave at 150 °C for 8 h. To collect precipitates, they were centrifuged numerous times at 3500 rpm for 10 min after cooling to the ambient temperature. To remove unreacted reactants, the precipitates were thoroughly washed with distilled water and ethanol. Drying the precipitates in an electric oven at 60 °C for 12 h was the final step. The powder was calcined at 800 °C for 2 h in a muffle furnace, forming Co_2_SnO_4_ nanocubes [40].

### 3.3. Synthesis of WS_2_

In order to create tungsten disulfide nanoparticles, 0.01 mol of sodium tungstate (Na_2_WO_4_·2H_2_O), 0.04 M of thiourea (CH_4_N_2_S), and 0.03 M of hydroxylamine hydrochloride (NH_2_OH·HCl) were combined in 120 mL of DI water and agitated for a few minutes using a magnetic stirrer. Subsequently, after a designated interval, we incorporate 0.50 g of CTAB (trimethyl ammonium bromide), which functions as a surfactant. The produced solution was agitated on a magnetic stirrer for approximately 1 h until a white precipitate formed at a specific pH. The white precipitate was subsequently transferred to a 150 mL Teflon-lined autoclave and subjected to a reaction furnace at 180 °C for 24 h. Following the cooling of the autoclave to room temperature, the contents were subjected to filtration and subsequently rinsed with deionized water and ethanol. The filtered sample was dried in a vacuum oven at 40 °C for approximately 4 h. Upon heating the sample, a progressive alteration in hue was noted, transitioning from light grey to dark grey. The sample was further crushed with a mortar and pestle, yielding fine-grained tungsten disulfide nanoparticles [3].

### 3.4. Synthesis of NHCSs

NHCSs were successfully synthesized using a previously reported method from the literature [29,67].

### 3.5. Synthesis of Co_2_SnO_4_/WS_2_@NHCSs

**Scheme 3:**
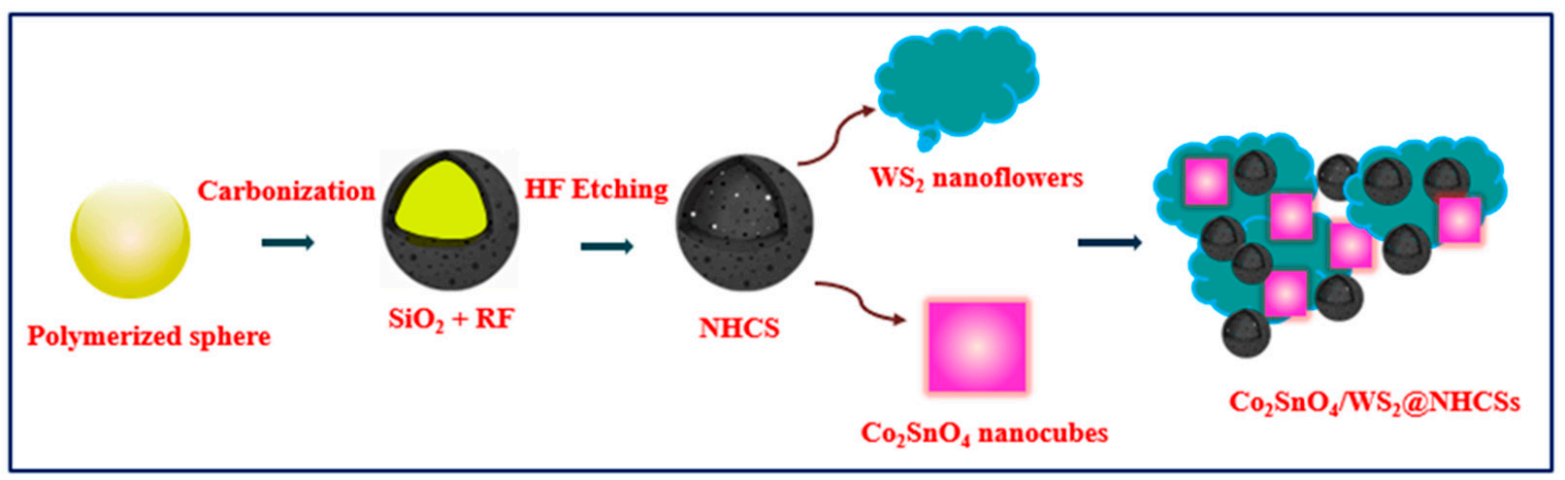
Schematic representation of the preparation of the Co_2_SnO_4_/WS_2_@NHCSs heterostructures photocatalysts.

The schematic representation of the synthesis process for the Co_2_SnO_4_/WS_2_@NHCSs nanocomposite is illustrated in Scheme 3.

### 3.6. Characterization Techniques

As part of an XPERTPRO multipurpose X-ray diffractometer study, X-ray diffraction (XRD) was used to look into the materials’ crystallinity and structural orientation. The shape of the catalyst was looked at with a Hitachi, S-4800, scanning electron microscope, (SEM, Hitachi, Ltd., Tokyo, Japan). The Japanese company Horiba Co., Ltd. made the Horiba EMAX machine (Horiba Co., Ltd., Tokyo, Japan), which was used to study the elemental compositions using Energy Dispersive X-ray Spectroscopy (EDS). A field-emission transmission electron microscope (FE-TEM, Tecnai F30 S-Twin, Hillsboro, OR, USA) was used to learn more about the nanocatalysts that were made. We used a single-color Al K-1486.6 eV source and X-ray photoelectron spectroscopy to look at the oxidation states of each element. With the help of the Kratos AXIS ULTRA DLD device (Kratos, Manchester, United Kingdom) this was performed. A UV-2450 Shimadzu spectrophotometer (Shimadzu, Kyoto, Japan) was used to figure out the bandgap of the materials by looking at the diffuse reflectance spectroscopy (DRS) spectra. An IR Tracer-100 device from Shimadzu (Kyoto, Japan) was used to look at the FT-IR spectra of the materials. The amount of Congo red was found using a Shimadzu UV-1800 UV-Vis spectrophotometer. A Vanquish TM UHPLC System (Thermo Fisher Scientific, Waltham, MA, USA) was used along with a Q Exactive quadrupole-electrostatic field Orbitrap high-resolution mass spectrometer (Thermo Fisher Scientific, Waltham, MA, USA) to find the parts of the breakdown that were found. A Hypersil GOLDTM a Q column (100 × 2.1 mm, 3 µ) (Thermo Fisher Scientific, Waltham, MA, USA) was used to sort the chemicals.

### 3.7. Photodegradation Process

A comparative analysis was conducted to assess the photodegradation characteristics of various photocatalytic materials, including Co_2_SnO_4_, WS_2_, NHCSs, Co_2_SnO_4_/WS_2_, Co_2_SnO_4_@NHCSs, WS_2_@NHCSs, and Co_2_SnO_4_/WS_2_@NHCSs heterostructures, in relation to the degradation of CR under visible-light irradiation (λ = 400–800 nm) from a 250 W Xenon arc lamp fitted with an ultraviolet (UV) cut-off filter. Thirty milligrams of each catalyst were individually introduced to 100 mL of dilute CR solution (10 mg/L of deionized water) and stirred in darkness for 30 min to achieve adsorption–desorption equilibrium. At 15 min intervals, 5 mL of the solution was extracted and centrifuged, and the supernatant was examined using a UV-Vis spectrometer to examine the variation in starting dye concentration. The absorbance spectra of the solutions before irradiation were recorded as well. The experiment was conducted in the presence of several scavengers, and recycling analysis was performed using the aforementioned approach. The subsequent formula was employed to determine the degradation efficiency:

D% = (C_0_ − C_t_)/C_0_ × 100% (4)

where C_0_ represents the concentration of the initial CR solution and C_t_ denotes the concentration of the CR solution following illumination at various time intervals. The absorption wavelength for CR is 496 nm.

## 4. Conclusions

In this work, we report the synthesis and detailed characterization of a novel ternary heterostructure, Co_2_SnO_4_/WS_2_@NHCSs, which shows promise as a high-tech photocatalyst for cleaning up polluted environments. The hydrothermal-calcination process was used for developing the heterostructures, which have unique optical and structural characteristics that made them very effective photocatalysts. The Co_2_SnO_4_/WS_2_@NHCSs ternary heterostructures demonstrated an impressive degradation efficiency of 87.22% for Congo red (CR) within only 60 min under visible-light irradiation, according to the photocatalytic degradation tests. The pristine catalyst samples were found to have rate constants 2.8-times lower than the greatest apparent rate constant of the Co_2_SnO_4_/WS_2_@NHCSs ternary heterostructures PCs (k = 0.0201 min^−1^ for CR dye). The NHCS spheres successfully formed Co_2_SnO_4_/WS_2_ heterostructures, which led to an increase in the visible-light absorption region and a decrease in the optical energy gap energy values from 1.76 eV to 1.72 eV, as compared to other catalysts. Superoxide radicals (^•^O_2_^−^) and hydroxide radicals (^•^OH) played crucial roles in the degradation process, as was further demonstrated by radical scavenging tests. The remarkable performance is a result of the innovative Z-scheme charge-transfer mechanism, which not only improves charge separation and reduces electron–hole recombination, but also provides a long-term, effective solution to the problem of water pollution. The reusability investigation highlights the possibility of using heterostructures of Co_2_SnO_4_ and WS_2_ with NHCSs as stable and highly efficient photocatalysts for real-world wastewater treatment applications. The industrial scalability and parameter refinement of Co_2_SnO_4_/WS_2_@NHCSs heterostructures, as well as their versatility and durability, should be the primary goals of future research. In general, the results of this study establish a basis for the fabrication of next-generation photocatalytic materials with an objective of environmentally responsible management, making a substantial contribution to the field of photocatalysis and environmental remediation.

## Figures and Tables

**Table 1:** Comparison studies of the kinetics parameters for the photocatalytic degradation of Congo red (CR) dye degradation under visible-light irradiation using various catalysts.

		Degradation Efficiency (%)	Apparent Rate Constants k (min^−1^)	
		CR	R^2^	k
1	Without Catalyst	0.9656	0.9656	2.187 × 10^−4^
2	Co_2_SnO_4_	0.9221	0.9221	0.0087
3	WS_2_	0.9739	0.9739	0.0099
4	NHCSs	0.9648	0.9648	0.0179
5	Co_2_SnO_4_@NHCSs	0.9109	0.9109	0.0131
6	WS_2_@NHCSs	0.8751	0.8751	0.0171
7	Co_2_SnO_4_/WS_2_	0.9259	0.9259	0.0102
8	Co_2_SnO_4_/WS_2_@NHCSs	0.9769	0.9769	0.0201

**Table 2:** The comparison study of WS_2_-based photocatalyst for the degradation of organic pollutants.

S. No	Catalyst	Wt. of Catalyst (g/L)	Organic Pollutant	Light Source	% of deg.	Deg. Time (min)	Ref.
1	WS_2_/BiOCl	0.05	MG	Visible light	94	180	[4]
2	WS_2_	0.1	BV 10	Ultrasonic	94.01	150	[44]
3	WS_2_/Polyaniline	0.02	MB	UV light	93	90	[59]
4	WS_2_/ZnIn_2_S_4_	0.025	MG	UV light	90	5	[60]
5	CdS/MoS_2_/WS_2_	0.01	RhB	Visible light	90	30	[61]
6	CdS/WS_2_	0.03	MB	Visible light	91	90	[62]
7	WS_2_/ZnO	0.05	MB	Visible light	80.5	120	[26]
8	Co_2_SnO_4_/WS_2_@NHCSs	0.03	CR	Visible light	87.22	60	This Work

MG— Malachite Green; MB—Methylene blue; RhB—Rhodamine B; CR—Congo red.

## Data Availability

The original contributions presented in this study are included in the article; further inquiries can be directed to the corresponding author.
